# Case Report: Basaloid Squamous Cell Carcinoma of the Tongue

**DOI:** 10.12688/f1000research.134826.3

**Published:** 2024-03-26

**Authors:** Samiha Jameel Ahmed Khan, Madhuri Gawande, Alka Hande, Swati Patil, Archana Sonone, Aayushi Pakhale

**Affiliations:** 1 Department of Oral and Maxillofacial Pathology and Microbiology, Sharad Pawar Dental College, Datta Meghe Institute of Higher Education and Research, Wardha, Maharashtra, 442001, India

**Keywords:** Basaloid squamous cell carcinoma, dimorphic pattern, basaloid cells, comedo necrosis ​​

## Abstract

Basaloid squamous cell carcinoma (BSCC) is a rare variant of conventional squamous cell carcinoma (SCC) frequently affecting the upper aerodigestive tract. The hypopharynx, tonsil, supraglottic larynx, tongue (base), and head-neck regions are particularly susceptible to BSCC. Clinically, the presentation of BSCC and conventional SCC is similar, but BSCC has a poorer prognosis. BSCC is distinguished histopathologically by a dimorphic pattern, a distinctive basal cell component paired with a squamous component. However, its similar features to conventional SCC makes it difficult to diagnose. Therefore, histopathology and immunohistochemistry play a crucial role in diagnosing such tumors. Here we present the case of a 70-year-old male diagnosed with BSCC involving the tongue.

## Introduction

Basaloid squamous cell carcinoma (BSCC) is a rare and aggressive form of oral squamous cell carcinoma (OSCC). Wain *et al.* ^1^ were the first to report the existence of BSCC, which was later proved to be a high-grade variety of SCC that is most common in the head and neck. ^2^ Males over the age of 50 are more likely to develop BSCC. It is regarded as a high-grade tumor with a higher risk of nodal metastasis (64%) and distant metastasis (44%), compared to conventional SCC. ^3^ The larynx and hypopharynx, which are parts of the upper-aerodigestive-tract, are often impacted. The tongue (base) is most frequently affected (61%), and BSCC is more common in the oral cavity to the rest of the body. The palate, the retromolar trigone, the gingival-mucosa, and the floor-of-the-mouth (30%) are other affected locations. ^4^^,^^5^ In terms of histopathology, the presence of solid epithelial cells with malignant characteristics and a basaloid appearance distinguishes BSCC the most. ^6^ The invading tumor exhibits a variation of development forms including cords and nests, trabeculae, cysts and glands. ^7^ Based on histopathologic and immunohistochemical findings, BSCC is distinct from conventional SCC. In addition, BSCC exhibits a different clinical behavior and prognosis than conventional SCC. ^8^ Compared to conventional SCC, the prognosis for BSCC is worse. Despite having different histological characteristics, BSCC is frequently misdiagnosed as neuroendocrine tumors, small cell carcinoma, adenosquamous carcinoma, and adenoid cystic carcinoma. ^9^^,^^10^ Here, we describe a case of BSCC in a 70-year-old man that affected the right lateral border of the tongue.

## Case report

A male patient aged 70 was referred to our Institute with a painful ulcer over the right lateral border of the tongue for two years. He also had pain that was dull type, continuous in nature, and non-radiating. He also complained of a burning sensation when eating spicy food. He was experiencing difficulty in mastication and deglutition, and the tongue movements were restricted. He experienced weight loss, loss of appetite, decreased salivation, and hoarseness of voice. The patient had a habit of kharra (smokeless tobacco) chewing two-three times a day for two years. He was also a chronic bidi smoker, from 20-25 years (two-three times per day). He claimed to have quit the habit ten years before.

**Figure 1.**
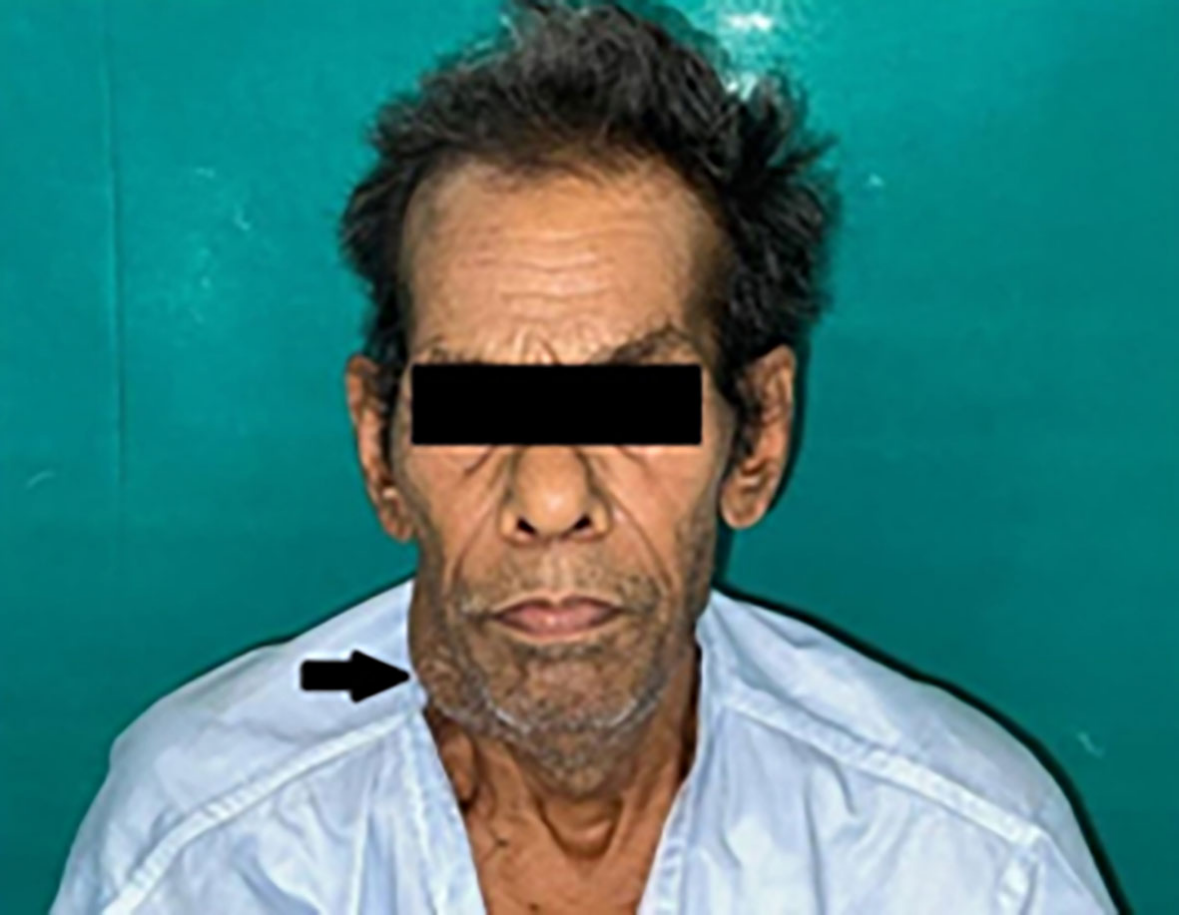
Extraoral photograph of the patient showing swelling on right side of the jaw.

Extra-oral findings revealed bilateral submandibular LN (lymph nodes), which were tender and palpable, measuring approximately 3 × 4 cm along its maximum dimension [ Figure 1].

**Figure 2.**
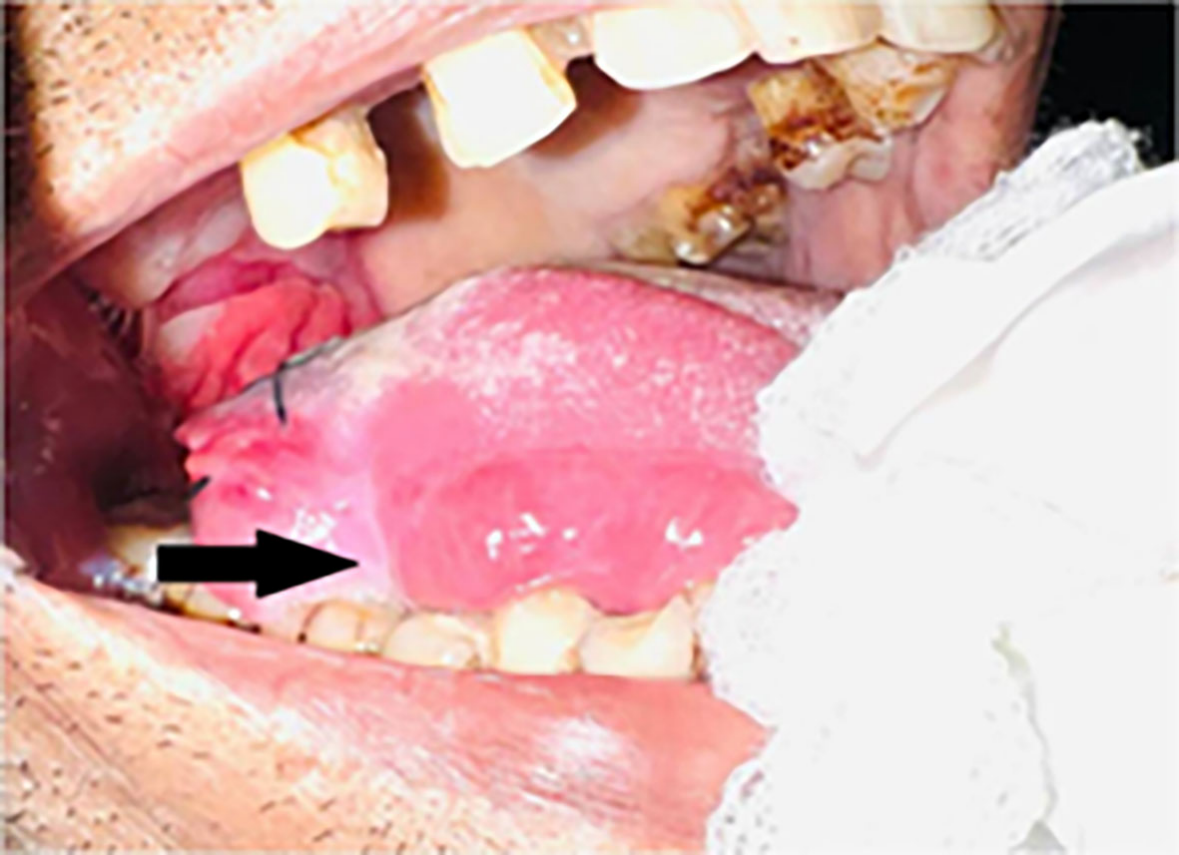
Clinical presentation of the case showing ulcero-proliferative lesion on right lateral border of tongue.

Intraoral examination revealed an ulceroproliferative lesion of approximately 2 × 3 cm on the lateral border of the tongue (right side) [ Figure 2], which was extending supero-inferiorly from the dorsal surface to the ventral surface of the tongue, anteroposteriorly from 46 region to the retromolar trigone (RMT) and involving soft palate. The lesion showed characteristic malignant features. The margins were everted and induration was present on palpation. A provisional diagnosis was made of malignancy of the tongue.

**Figure 3.**
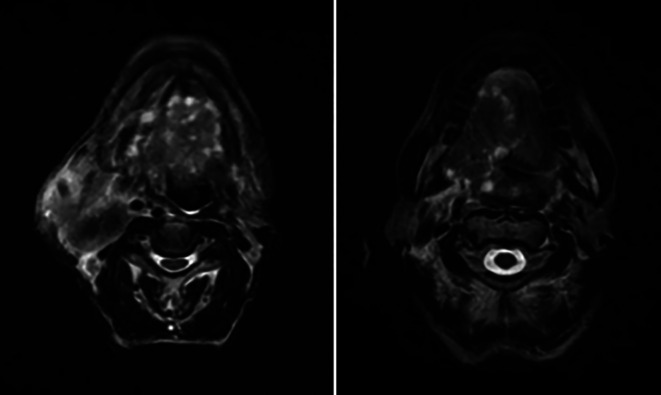
MRI (tongue) showing features mentioned above.

Further, a tongue MRI with contrast was performed, which showed a heterogeneously enhancing mass lesion on the tongue (right side) with areas of necrosis within, abutting the lingual septum medially and extending into the infratemporal fossa laterally measuring approximately 7.6 × 4.5 × 4.3 cm. There was evidence of multiple subcentimetric to centimetric heterogeneously enhancing lymph nodes in the submental, bilateral submandibular, and jugulodigastric region, the largest being 4.3 × 3.2 cm in size in the right submandibular region with necrotic areas within. Impression of the tongue MRI revealed the abovementioned characteristics, suggesting tongue carcinoma with lymphadenopathy [ Figure 3].

An incisional biopsy was done at our institute. The details of the biopsy report are mentioned below.

### Histopathological and immunohistochemical report

**Figure 4.**
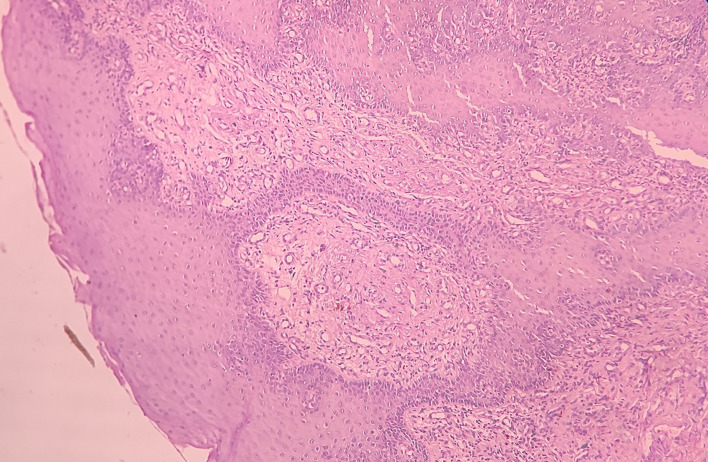
Haematoxylin and eosin-stained tissue section at 4× (scanner view) showing hyperplastic stratified squamous epithelium and fibro cellular CT stroma.

Haematoxylin and eosin-stained tissue section revealed an overlying hyperplastic parakeratinizing stratified squamous epithelium and underlying fibro cellular connective tissue (CT) stroma [ Figure 4].

**Figure 5.**
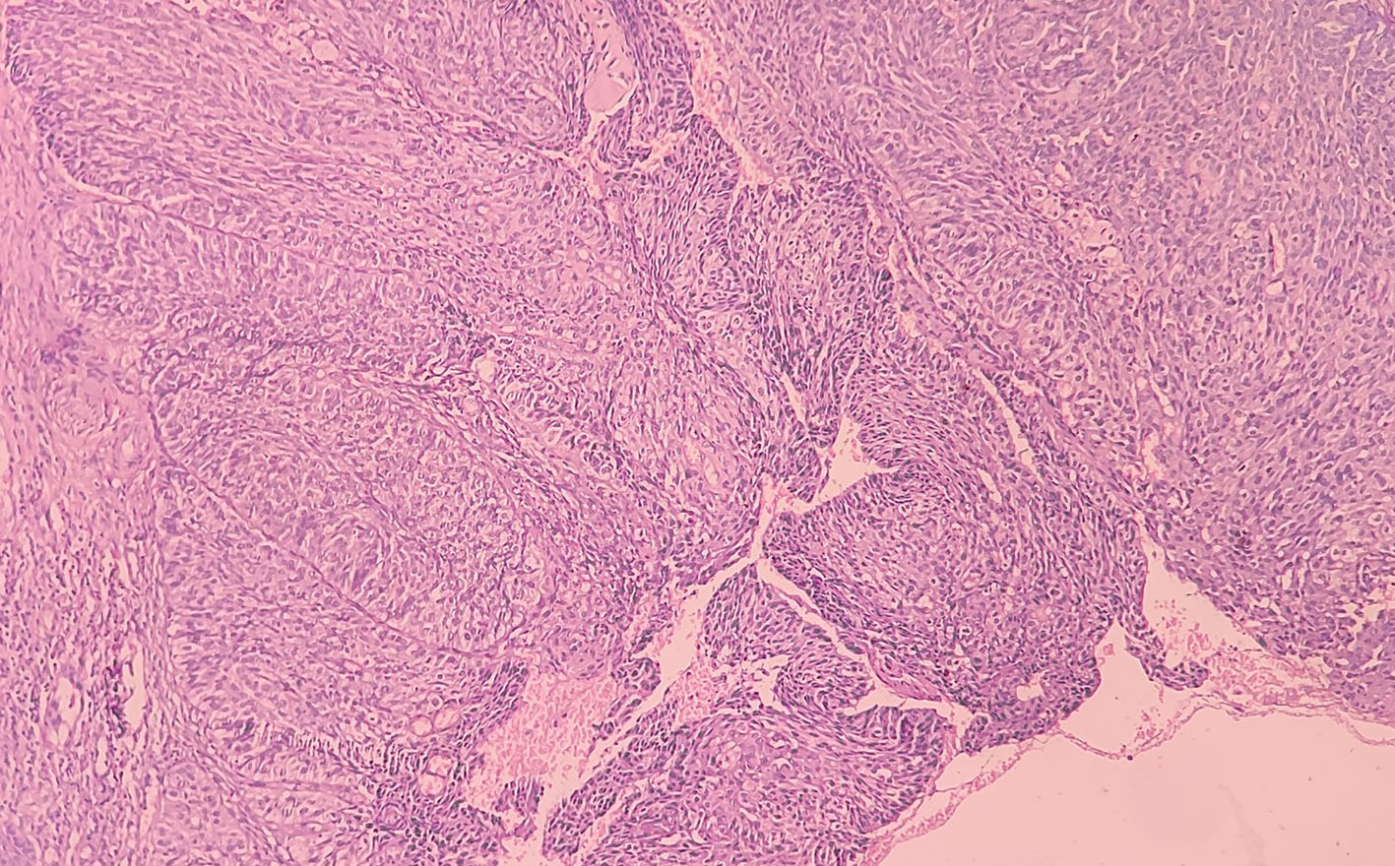
Haematoxylin and eosin-stained tissue section at 10× (low power view) showing islands of squamous cells with basaloid component.

At low-power view [ Figure 5], it was evident that the epithelial cells invaded the CT in the form of islands. Some of these islands consisted of basaloid and squamous cells. These islands showed cystic spaces with a central area of comedo-necrosis. There was presence of malignant epithelial cells arranged in an organoid pattern displaying lobules of neoplastic epithelial cells. Fibrous CT septa separated these cells. The tumor cells were compactly arranged and showed cellular pleomorphism. The CT were comprised of collagen fibers and a few fibroblasts. Numerous endothelial cells-lined blood vessels with intravasated and extravasated red blood cells (RBCs) were seen. Moderate to chronic inflammatory cell infiltrates were also seen.

**Figure 6.**
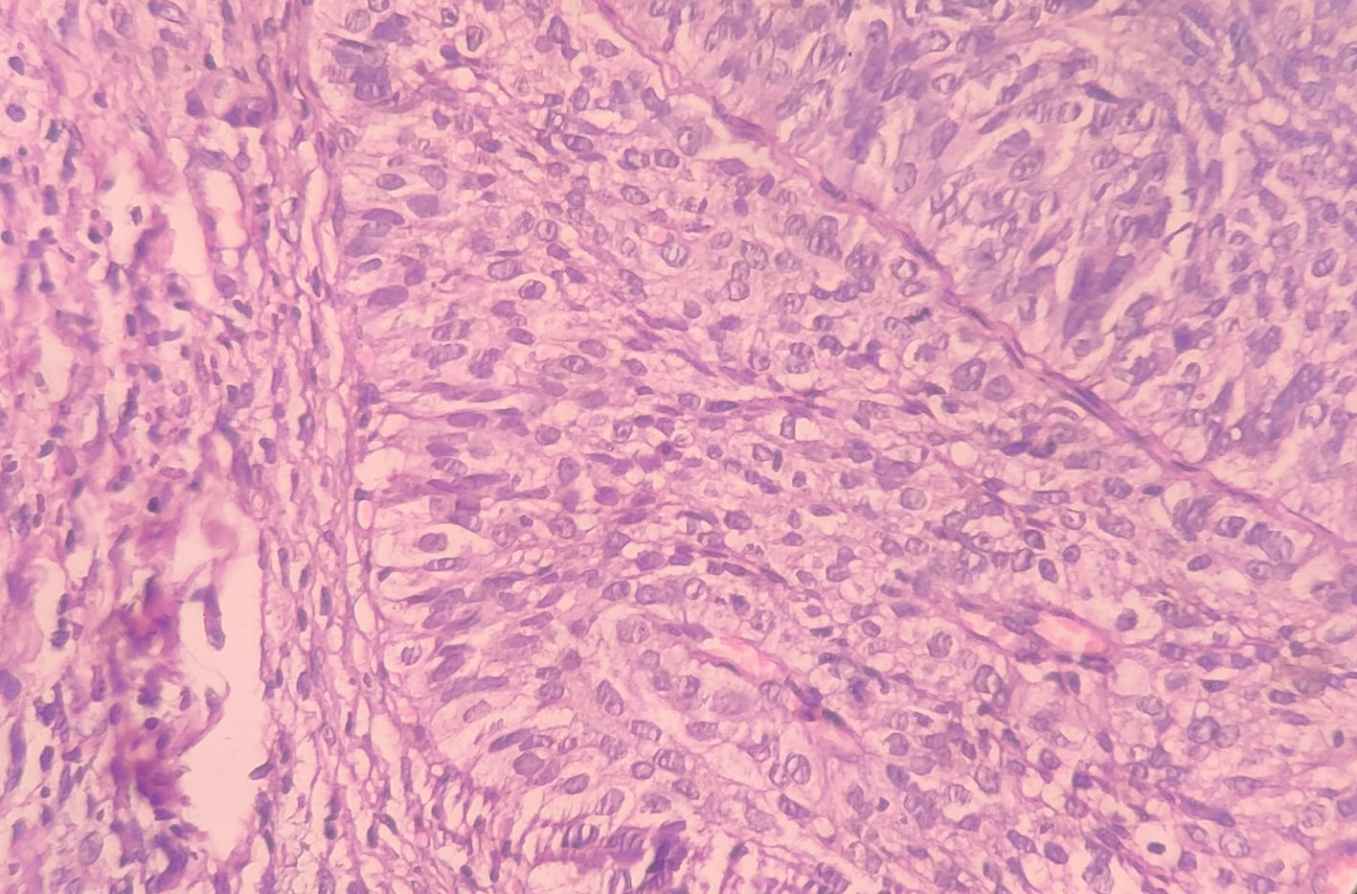
Haematoxylin and eosin-stained tissue section at 40× (high power view) showing hyperchromatic nuclei with nuclear palisading.

**Figure 7.**
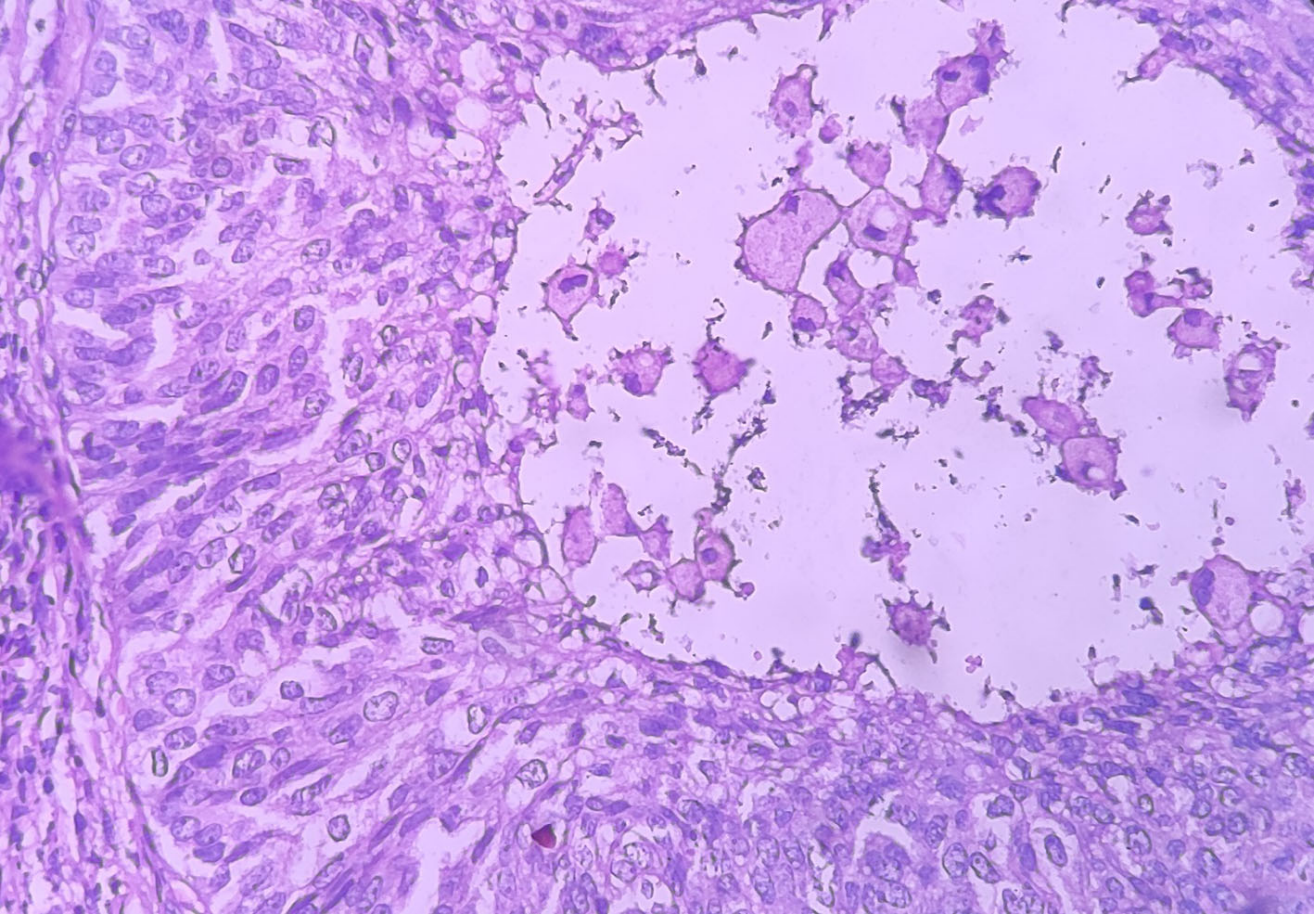
Haematoxylin and eosin-stained tissue section showing cystic space with central area of comedo-necrosis.

Under the high-power view [ Figures 6, 7], all findings of the low power view were confirmed. The periphery of neoplastic islands showed cuboidal to low-columnar basaloid cells with palisading nuclei. The nuclei were ovoid-shaped, showing nuclear hyperchromatism and scant cytoplasm. The neoplastic cells showed characteristics like cellular pleomorphism, nuclear pleomorphism and hyperchromatism; there was increase in the nuclear-cytoplasmic ratio, and abnormal mitosis was also evident. There was presence of chronic inflammatory cell infiltrate chiefly comprising of lymphocytes.

## Discussion

BSCC, a distinct variation of conventional SCC, more commonly affects males in the age group of 60 to 70. ^11^ Wain *et al*. ^1^ originally identified BSCC as an uncommon, histologically different, and extremely aggressive subtype of SCC in 1986. These four main histologic characteristics were used to make the diagnosis of BSCC: (a) cells present in solid groups in a lobular arrangement, close to the surface mucosa; (b) small, densely packed cells with scant cytoplasm; (c) dark/hyperchromatic nuclei without nucleoli; and (d) small, cystic spaces consisting of mucin-like material. ^12^ BSCC was initially noted in the oral cavity by Cadier and others. The literature has described 45 instances of BSCC affecting the oral cavity, with the base of the tongue (61%) and floor of the mouth (30%) showing a strong preference. ^6^^,^^13^

Similar to our case, BSCC is said to be more common in older age groups. ^14^ However, compared to conventional SCC, several investigations have indicated an equal frequency in both sexes. ^15^ In terms of etiology and pathology, basaloid SCC is comparable to conventional SCC. The majority of BSCC patients are seen to have a long history of alcohol and tobacco consumption.

Similar to conventional SCC, BSCC has a painless irregular mass that is hard, verrucous or smooth, ^15^ and may or may not be ulcerative. ^14^^,^^16^^–^^18^ Because of this, it is quite challenging to distinguish it from conventional SCC. Therefore, the histopathologic and immunohistochemical characteristics play a major role in the diagnosis. ^8^ It can be difficult for a pathologist to diagnose BSCC using an incisional biopsy since BSCC shares many histological characteristics with other neoplasms that have a similar microscopic appearance.

Basal-cell-carcinoma, adenoid-cystic carcinoma (solid variety), adeno-squamous carcinoma, basal-cell adenocarcinoma, salivary-duct carcinoma, and neuro-endocrine carcinoma are all included in the differential diagnosis for BSCC.

The solid type of adenoid cystic carcinoma (ACC) is characterized by clusters of cuboidal cells with black nuclei. ^19^ Most ACCs respond with antibodies to CD117; squamous differentiation, cytologic atypia, and nuclear atypia are absent from ACCs. ^20^ CD117 is used to distinguish between ACCs (solid variant) and BSCCs since ACCs show a positive CD117 test whereas BSCCs do not. Basaloid, columnar, or mucin-secreting cells line the glandular structures seen in adenosquamous carcinomas. Mucicarmine staining demonstrates intracytoplasmic mucin. ^19^

Small round cells and giant polygonal cells are the two cell types commonly mixed together in basal cell adenocarcinomas. In order to diagnose cancer, more than four to five mitotic figures per ten high-power fields are required. ^19^

In cases of basal cell ameloblastoma, homogeneous basaloid-appearing cells surround core islands of odontogenic epithelium, which are periphery surrounded by cuboidal cells. Both the squamous component and central comedo necrosis are absent. ^19^

Tumour islands with extensive centre cystic gaps and comedo necrosis are observed in salivary duct carcinoma. The peripheral tumour cells are cuboidal/polygonal and contain a modest proportion of eosinophilic cytoplasm. The tumour cells are several cell layers thick. ^19^

When compared to the others, adenoid cystic carcinoma (ACC) most closely resembles BSCC. According to Klijanienko *et al*., it can be challenging or impossible to distinguish between BSCC and ACC, particularly in incisional biopsies. Clinically, BSCC is thought to be more destructive than conventional SCC. ^10^ In comparison with conventional SCC, BSCC has a worse prognosis and survival rate. Less than half as many BSCC patients survive compared to those with conventional SCC. ^18^

Positive staining of cyto-keratin 13 (CK-13) in the well-differentiated-squamous cells distinguishes SCC from BSCC, however the majority of basaloid cells in BSCC does not exhibit immunoreactivity. ^8^ In a study by Ricardo *et al*., ^17^ it was discovered that BSCC has higher levels of the PCNA (proliferating-cell nuclear-antigen), AgNOR (argyrophilic-nucleolar-organizing region), and p53 protein than SCC did. Additionally, matrix megalloproteins (MMP-1, 2 and 9) expression levels were observed to be higher in BSCC than in SCC, stating that BSCC exhibits a more destructive/aggressive behavior than SCC. ^6^

While distant metastasis is around six times higher in BSCC than in conventional SCC, local recurrences are less common. ^5^ In contrast to just 13% of conventional SCC, Winzenburg *et al*. reported 52% distant metastasis of BSCC. ^21^

There isn’t a definite treatment consensus. The majority of the literature has recommended radiotherapy combined with surgery to remove the tumour and lymph nodes as the initial course of treatment. ^22^ Concurrent chemoradiotherapy (CCRT) was used as the main form of treatment in our situation.

Although there is still a lot of disagreement on how to compare the clinical trajectory and prognosis of BSCC and conventional SCC. It has been established that BSCC is a worse version of conventional SCC. Compared to SCC, it has a worse prognosis and a higher recurrence rate. In order to better understand and distinguish unusual lesions like BSCC from conventional SCC and improve therapy and prognosis, it is necessary to report them.

## Conclusion

BSCC is a rare and aggressive variant of squamous cell carcinoma. Because of its aggressive nature, it is crucial to identify the disease at an early stage. Histopathology and immunohistochemistry play an important role in the diagnosis of BSCC and to differentiate it from conventional SCC. Appropriate treatment should be followed after the diagnosis considering its aggressiveness and high rate of metastasis.

## Consent

Prior written informed consent was obtained from the patient and other individuals involved in the study.

## Data Availability

All data underlying the results are available as part of the article and no additional source data are required. Zenodo: Figure 4, https://doi.org/10.5281/zenodo.7944356. ^23^ Zenodo: Figure 5, https://doi.org/10.5281/zenodo.7944418. ^24^ Zenodo: Figure 6, https://doi.org/10.5281/zenodo.7944429. ^25^ Zenodo: Figure 7, https://doi.org/10.5281/zenodo.7944441. ^26^ Zenodo: CARE checklist for Case Report: Basaloid Squamous Cell Carcinoma, https://doi.org/10.5281/zenodo.7902239. ^27^ Data are available under the terms of the Creative Commons Attribution 4.0 International license (CC-BY 4.0).
