# Supplementary material for: Case Report: Basaloid Squamous Cell Carcinoma of the Tongue
Source: F1000Res. 2024 Mar 26;12:1008. Originally published 2023 Aug 21. [Version 3] doi: 10.12688/f1000research.134826.3 (PMC10955190; doi:10.12688/f1000research.134826.3)
Supplement: Peer review history

# Reviewer report: Reviewer response for version 3

**DOI:** 10.5256/f1000research.164104.r261917

**Published:** 2024-04-30

**Authors:** Neetu Jain, Shashi Keshwar

**Affiliations:** 1 B.P. Koirala Institute of Health Sciences, Dharan, Nepal; 2 Oral Pathology, B.P. Koirala Institute of Health Sciences, Dharan, Koshi, Nepal

To

 The editorial Team

     I want to thank the editorial team and the author for considering me for this review. It is a very well written case report with all the details provided. It is really very important to report such cases to add value to medical literature.

    While the manuscript provides valuable insights, there are a few areas that could be refined to further augment the quality and impact of the work. I have mentioned some respectful suggestions that could potentially improve the manuscript if the author chose to implement them.

 1. In introduction, WHO definition and categorization for Basaloid squamous cell carcinoma can be kindly added.

 2. On Page no. 3 in case report session, the 1st line mentions that the patient had lesion since 2 years, it will be more informative if mentioned whether the lesion had any progressive course over a certain period of time or is it the same since two years.

 3. As per the figure-1, the swelling seems unilateral, so the size of the enlarged sub-mandibular lymph nodes must be more on this side. So kindly correct that.

 4. In figure-2 the arrow has been placed in an opposite direction, as its mentioned that the extension of the lesion is from 46 to retro molar trigone. Kindly correct it.

 5. In figure-5 and 6, it will be more effective if the significant areas are highlighted with arrows, like-basaloid component, squamous component, palisading, comedo necrosis and abnormal mitotic activity.

 6. The color of the figure-7 needs to be adjusted or kindly mention if some other stains were used to show the comedo necrosis.

 7. On page -5 its mentioned histopathological and immunohistochemical report, so if any immunohistochemical tests are also done, kindly add the details of that as well.

 8. In paragraph 5 of page-7, its mentioned black nuclei. Does it mean dark nuclei? Kindly correct it.

 9.In paragraph-6 of page-7, its better to be more specific. so kindly correct the line instead of cancer, mention it as basal cell adenocarcinoma.

 10.In page-7, paragraph-7, kindly re-frame the sentence. As in case of Basal cell ameloblastoma, the epithelial islands have basaloid cells at the center of the island surrounded by peripheral cuboidal cells.

 11.In page 7, paragraph-8, cystic spaces is a more scientifically used term instead of cystic gaps, so kindly correct it.

 12. In page-7, paragraph-9: the first two lines can be shifted at the beginning of paragraph -5 of the same page. As this paragraph mentions the comparison between ACC and BSCC.

 These are some of the suggestions, I have mentioned if the author likes to address.

  Thanks and Regards

  Dr. Neetu Jain

Are enough details provided of any physical examination and diagnostic tests, treatment given and outcomes?

Partly

Is the case presented with sufficient detail to be useful for other practitioners?

Partly

Is sufficient discussion included of the importance of the findings and their relevance to future understanding of disease processes, diagnosis or treatment?

Yes

Is the background of the case’s history and progression described in sufficient detail?

Partly

Reviewer Expertise:

Oral Pathology, specially oral cancers.

We confirm that we have read this submission and believe that we have an appropriate level of expertise to confirm that it is of an acceptable scientific standard, however we have significant reservations, as outlined above.

# Reviewer report: Reviewer response for version 2

**DOI:** 10.5256/f1000research.159091.r255682

**Published:** 2024-03-20

**Authors:** Namrata Patil

**Affiliations:** 1 Saraswati Dhanwantari Dental College and Hospital, Parawa, Maharashtra, India

The manuscript provides a valuable insight on the oral Busloid squamous cell carcinoma which is rarely encountered in routine examination. Overall a satisfactory article. this article is providing very good observation and data in the field of oral pathology and microbiology. This data is very important for future studies. This is nice.

Are enough details provided of any physical examination and diagnostic tests, treatment given and outcomes?

Yes

Is the case presented with sufficient detail to be useful for other practitioners?

Yes

Is sufficient discussion included of the importance of the findings and their relevance to future understanding of disease processes, diagnosis or treatment?

Yes

Is the background of the case’s history and progression described in sufficient detail?

Yes

Reviewer Expertise:

oral pathology, molecular biology oral cancer

I confirm that I have read this submission and believe that I have an appropriate level of expertise to confirm that it is of an acceptable scientific standard.

# Reviewer report: Reviewer response for version 2

**DOI:** 10.5256/f1000research.159091.r226677

**Published:** 2023-12-18

**Authors:** Muhammad Kashif

**Affiliations:** 1 Department of Oral Pathology, Bakhtawar Amin Medical and Dental College, Multan, Pakistan

Case report could be more interesting if immunohistochemical staining had been performed as the discussion and conclusion sections are more focused on comparison of BSCC with other malignant tumours based on IHC markers profiling.

Are enough details provided of any physical examination and diagnostic tests, treatment given and outcomes?

Partly

Is the case presented with sufficient detail to be useful for other practitioners?

Partly

Is sufficient discussion included of the importance of the findings and their relevance to future understanding of disease processes, diagnosis or treatment?

No

Is the background of the case’s history and progression described in sufficient detail?

Yes

Reviewer Expertise:

HNSCC, Oral Pathology, Oral Immunology, Tumour Immunology

I confirm that I have read this submission and believe that I have an appropriate level of expertise to confirm that it is of an acceptable scientific standard.

# Reviewer report: Reviewer response for version 1

**DOI:** 10.5256/f1000research.147911.r207241

**Published:** 2023-10-12

**Authors:** Muhammad Kashif

**Affiliations:** 1 Department of Oral Pathology, Bakhtawar Amin Medical and Dental College, Multan, Pakistan

1. The title seems too much generic in its current form.
2. The author used three terms for OSCC "conventional", "traditional", "typical". Please be consistent with a single term and the suitable one is "conventional".
3. What is "RMT"? Define abbreviations in its first use.
4. How have the authors declared tongue carcinoma just on MRI findings? Clarify it.
5. In figure 4, I am unable to identify any type dysplasia in overlying squamous epithelium. It is just a hyperplastic epithelium.
6. Did authors performed immunohistochemistry? If no, then why did they discussed it in discussion section?
7. Discussion needs to rephrased on logical grounds with comparison of findings of this case with already published data. Currently, it seems merely an introduction section.
8. Add conclusion section.

Are enough details provided of any physical examination and diagnostic tests, treatment given and outcomes?

Partly

Is the case presented with sufficient detail to be useful for other practitioners?

Partly

Is sufficient discussion included of the importance of the findings and their relevance to future understanding of disease processes, diagnosis or treatment?

No

Is the background of the case’s history and progression described in sufficient detail?

Yes

Reviewer Expertise:

HNSCC, Oral Pathology, Oral Immunology, Tumour Immunology

I confirm that I have read this submission and believe that I have an appropriate level of expertise to confirm that it is of an acceptable scientific standard, however I have significant reservations, as outlined above.

# Response

**Published:** 2023-11-18

**Authors:** Samiha Khan

**Affiliations:** Oral Pathology, Sharad Pawar Dental College, Datta Meghe Institute of Higher Education and Research, Wardha, Wardha, Maharashtra, India

1. The title has been changed to Basaloid Squamous Cell Carcinoma of the Tongue: A Case Report

 2. The term conventional SCC has been implied in place of typical and traditional SCC.

 3. RMT: Retromolar trigone. Full form is mentioned in the revised manuscript.

 4. The MRI reports showed a heterogenously enhancing mass lesion on the tongue with areas of necrosis within. Lymphadenopathy was also seen. Based on clinical and radiological correlation, the diagnosis was made of tongue carcinoma.

 5. The epithelium is hyperplastic and changes have been made.

 6. Immunohistochemistry (IHC) was not performed in our case. However, IHC plays a crucial role in differentiating BSCC from conventional SCC. Hence, we have mentioned IHC results and reports from different authors in the literature.

 7. Few changes have been made in the discussion. New data is added according to case reports from different authors. 

 8. Conclusion is added.
